# Supplementary material for: On the estimation of genome-average recombination rates
Source: Genetics. 2024 Apr 3;227(2):iyae051. doi: 10.1093/genetics/iyae051 (PMC11232287; doi:10.1093/genetics/iyae051)
Supplement: iyae051_Supplementary_Data [file iyae051_supplementary_data.zip › Supplemental_Figure_11_GENETICS-2024-306814.pdf]

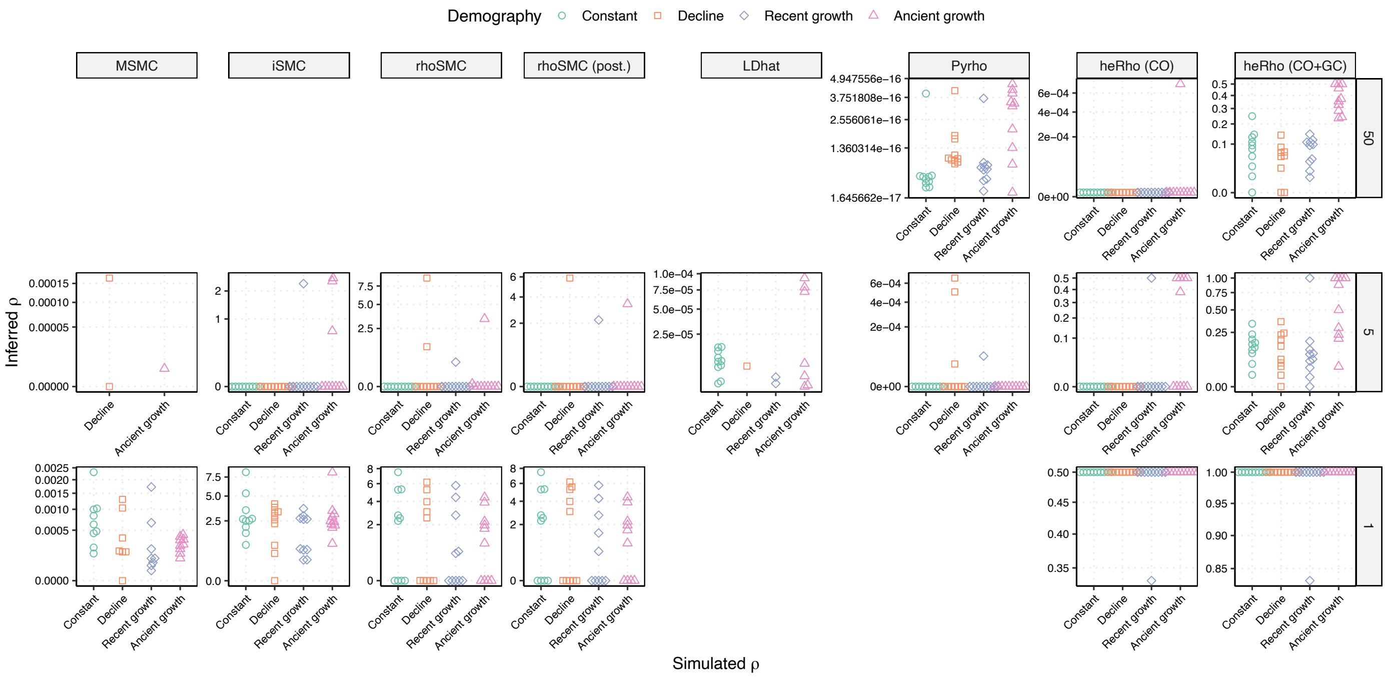

Supplementary Figure 11 Inference of the genome-wide population recombination rate when the simulated rate is zero. *LDhat* did not converge after 48h when used with 50 diploids and was stopped, so no estimate is reported. Several replicates did not converge either under the "decline" and "growth" scenarios when five diploids were used. Missing data for the *MSMC* method correspond to runs where the program failed to converge.
