# Supplementary material for: On the estimation of genome-average recombination rates
Source: Genetics. 2024 Apr 3;227(2):iyae051. doi: 10.1093/genetics/iyae051 (PMC11232287; doi:10.1093/genetics/iyae051)
Supplement: iyae051_Supplementary_Data [file iyae051_supplementary_data.zip › Supplemental_Figure_12_GENETICS-2024-306814.pdf]

Supplementary Figure 12 (next page) Demography inference using MSMC in the presence of gene conversion. Ten datasets of five diploid individuals were simulated for each proportion of gene conversion events; under four demographic scenarios (dashed lines) and a heterogeneous recombination landscape. Column facets depict distinct amounts of gene conversion events, and row facets compare the inference methods: default parameters (top) or reduced time intervals (bottom). Framed numbers show the proportion (out of 10) of replicates where the optimization converged.

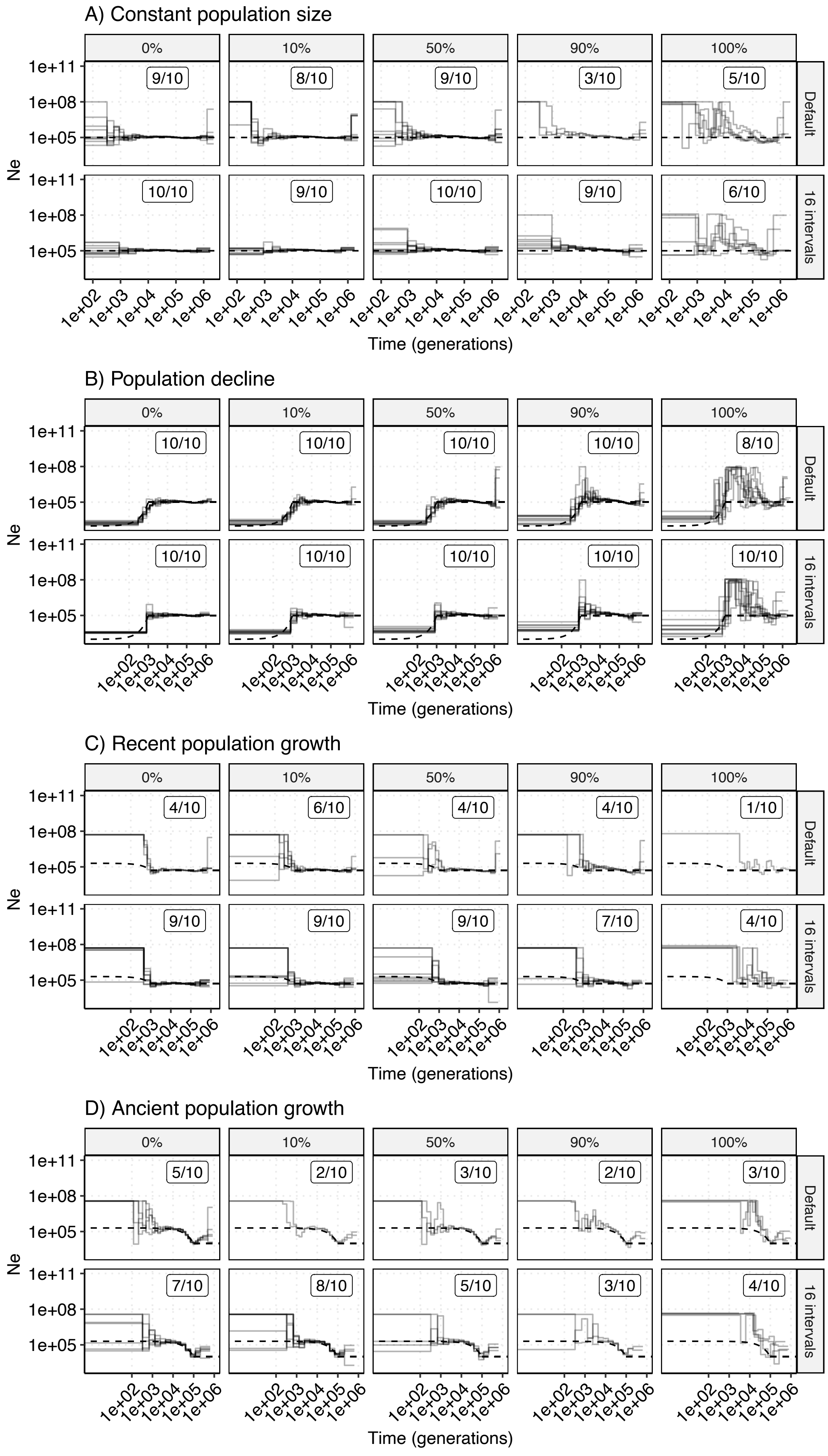
A) Constant population size
0%
10%
50%
90%
100%
Default
16 intervals
9/10
8/10
9/10
3/10
5/10
10/10
9/10
10/10
9/10
6/10
B) Population decline
10/10
10/10
10/10
10/10
8/10
10/10
10/10
10/10
10/10
10/10
C) Recent population growth
4/10
6/10
4/10
4/10
1/10
9/10
9/10
9/10
7/10
4/10
D) Ancient population growth
5/10
2/10
3/10
2/10
3/10
7/10
8/10
5/10
3/10
4/10
Ne
1e+11
1e+08
1e+05
1e+02
1e+03
1e+04
1e+05
1e+06
Time (generations)
