# Supplementary material for: On the estimation of genome-average recombination rates
Source: Genetics. 2024 Apr 3;227(2):iyae051. doi: 10.1093/genetics/iyae051 (PMC11232287; doi:10.1093/genetics/iyae051)
Supplement: iyae051_Supplementary_Data [file iyae051_supplementary_data.zip › Supplemental_Figure_1_GENETICS-2024-306814.pdf]

Supplementary Figure 1 (next page) Demography inference using MSMC. Ten datasets of five diploid individuals were simulated for each recombination rate (here constant along the genome) under four demographic scenarios (dashed lines). Column facets depict distinct recombination rates, and row facets compare the inference methods: default parameters (top) or reduced time intervals (bottom). Each line represents the variation of the estimated population sizes in each time interval as a function of time before present (on the left), for each replicate where the model successfully converged. Framed numbers show the proportion (out of 10) of replicates where the optimization converged.

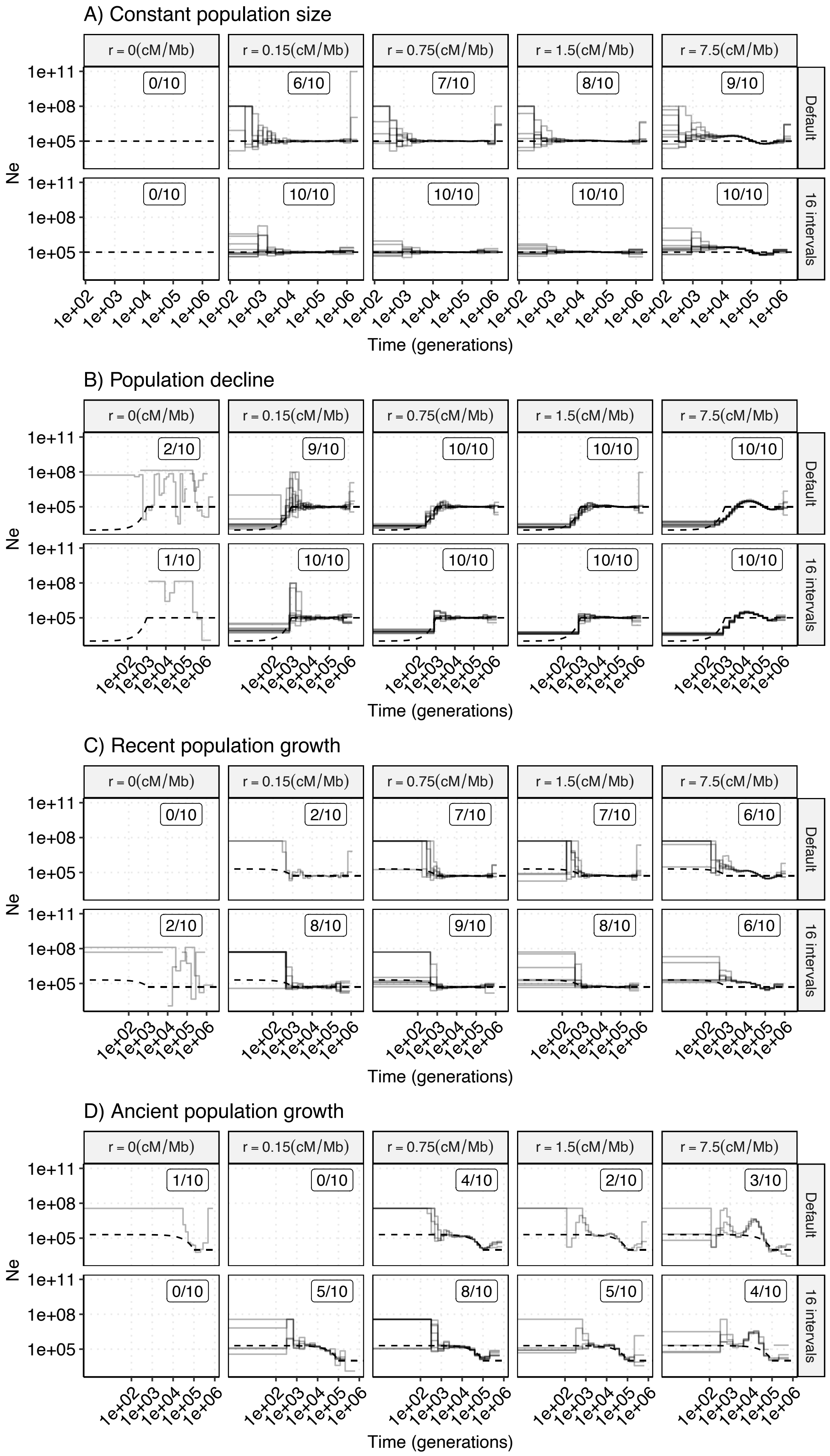
A) Constant population size
B) Population decline
C) Recent population growth
D) Ancient population growth
r = 0(cM/Mb)
r = 0.15(cM/Mb)
r = 0.75(cM/Mb)
r = 1.5(cM/Mb)
r = 7.5(cM/Mb)
Default
16 intervals
Ne
Time (generations)
1e+05
1e+08
1e+11
1e+02
1e+03
1e+04
1e+05
1e+06
0/10
6/10
7/10
8/10
9/10
0/10
10/10
10/10
10/10
10/10
2/10
9/10
10/10
10/10
10/10
1/10
10/10
10/10
10/10
10/10
0/10
2/10
7/10
7/10
6/10
2/10
8/10
9/10
8/10
6/10
1/10
0/10
4/10
2/10
3/10
0/10
5/10
8/10
5/10
4/10
