# Supplementary material for: On the estimation of genome-average recombination rates
Source: Genetics. 2024 Apr 3;227(2):iyae051. doi: 10.1093/genetics/iyae051 (PMC11232287; doi:10.1093/genetics/iyae051)
Supplement: iyae051_Supplementary_Data [file iyae051_supplementary_data.zip › Supplemental_Figure_2_GENETICS-2024-306814.pdf]

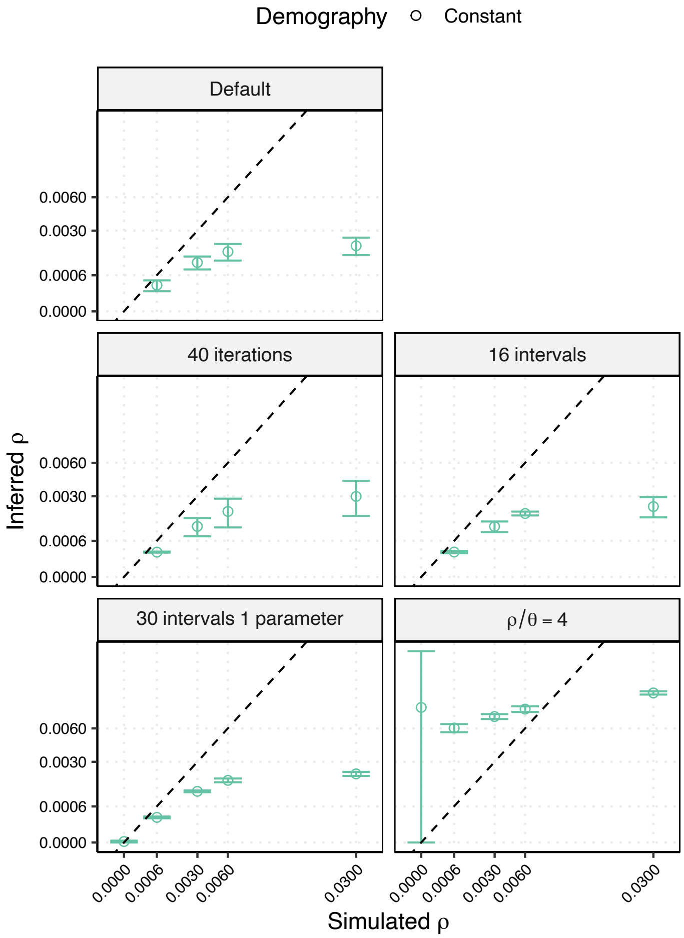

Supplementary Figure 2 Impact of the MSMC model and optimization procedure on the genome-average population recombination rate inference. Five diploid individuals were used in each simulated dataset. Missing points at $x = 0$ indicate that *MSMC* failed to converge. Legend as in Figure 1.
