# Supplementary material for: On the estimation of genome-average recombination rates
Source: Genetics. 2024 Apr 3;227(2):iyae051. doi: 10.1093/genetics/iyae051 (PMC11232287; doi:10.1093/genetics/iyae051)
Supplement: iyae051_Supplementary_Data [file iyae051_supplementary_data.zip › Supplemental_Figure_3_GENETICS-2024-306814.pdf]

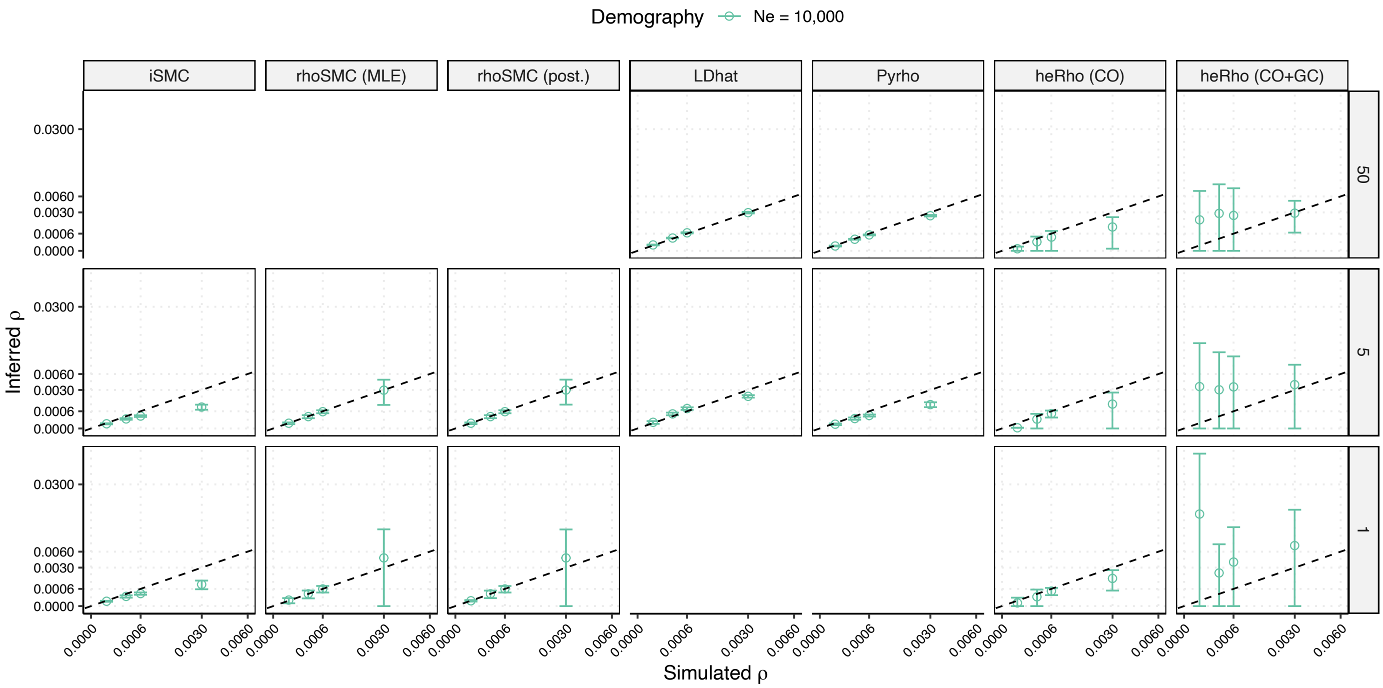

Supplementary Figure 3 Inference of the genome-wide population recombination rate under a heterogeneous recombination landscape and constant population size equal to 10,000 individuals. Legend as in Figure 2, which used a population size of 100,000 individuals.
