# Supplementary material for: On the estimation of genome-average recombination rates
Source: Genetics. 2024 Apr 3;227(2):iyae051. doi: 10.1093/genetics/iyae051 (PMC11232287; doi:10.1093/genetics/iyae051)
Supplement: iyae051_Supplementary_Data [file iyae051_supplementary_data.zip › Supplemental_Figure_4_GENETICS-2024-306814.pdf]

Supplementary Figure 4 (next page) Demography inference using MSMC. Ten datasets of five diploid individuals were simulated for each average population recombination rate under four demographic scenarios (dashed lines) and a heterogeneous recombination landscape. Column facets depict distinct recombination rates, and row facets compare the inference methods: default parameters (top) or reduced time intervals (bottom). Framed numbers show the proportion (out of 10) of replicates where the optimization converged.

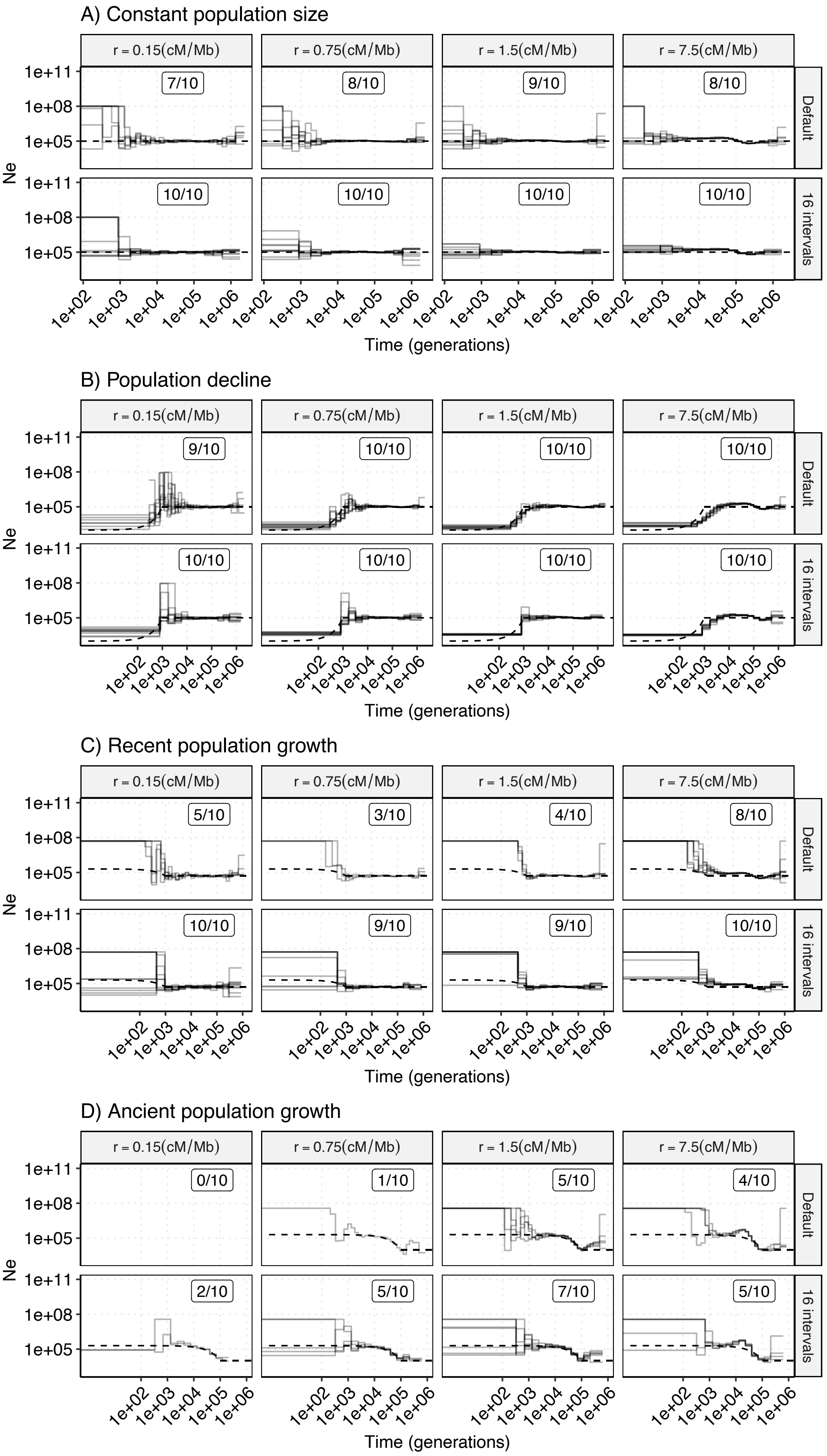
A) Constant population size
B) Population decline
C) Recent population growth
D) Ancient population growth
r = 0.15(cM/Mb)
r = 0.75(cM/Mb)
r = 1.5(cM/Mb)
r = 7.5(cM/Mb)
Default
16 intervals
Ne
Time (generations)
1e+11
1e+08
1e+05
1e+02
1e+03
1e+04
1e+05
1e+06
7/10
8/10
9/10
8/10
10/10
10/10
10/10
10/10
9/10
10/10
10/10
10/10
10/10
10/10
10/10
10/10
5/10
3/10
4/10
8/10
10/10
9/10
9/10
10/10
0/10
1/10
5/10
4/10
2/10
5/10
7/10
5/10
