# Supplementary material for: On the estimation of genome-average recombination rates
Source: Genetics. 2024 Apr 3;227(2):iyae051. doi: 10.1093/genetics/iyae051 (PMC11232287; doi:10.1093/genetics/iyae051)
Supplement: iyae051_Supplementary_Data [file iyae051_supplementary_data.zip › Supplemental_Figure_6_GENETICS-2024-306814.pdf]

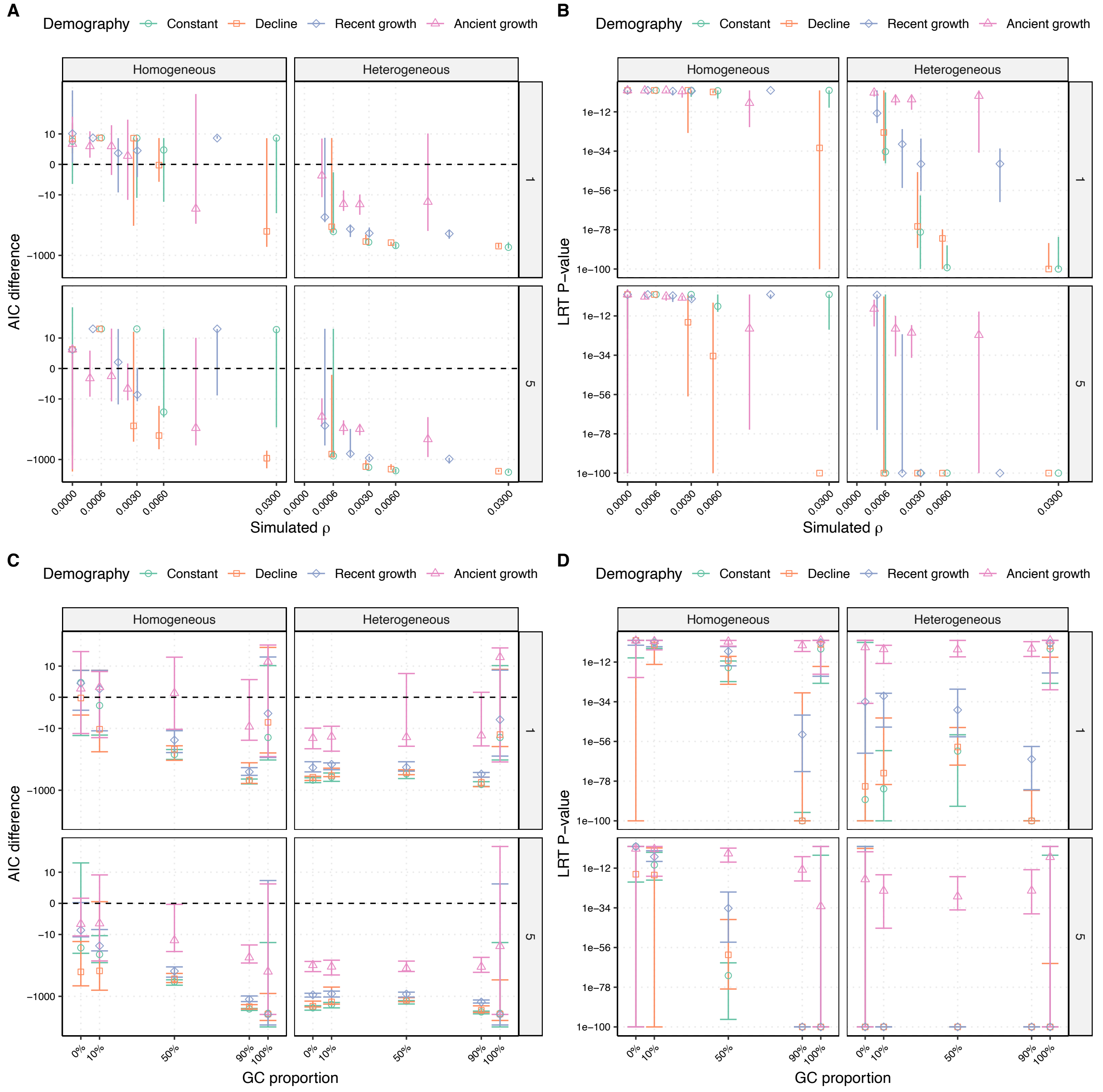

Supplementary Figure 6 Model comparisons on homogeneous and non-homogeneous recombination landscapes, with and without gene conversion. Column facets: simulations under a homogeneous (flat) or heterogeneous (variable) recombination landscape. Row facets: number of diploid individuals used for inference. A, B: impact of the recombination rate. C, D: impact of the proportion of gene conversion (GC). A, C: Akaike's information criterion (AIC). B, D: likelihood ratio test (LRT). Median (points), minimal and maximal values (error bars) over ten replicates.
