# Supplementary material for: On the estimation of genome-average recombination rates
Source: Genetics. 2024 Apr 3;227(2):iyae051. doi: 10.1093/genetics/iyae051 (PMC11232287; doi:10.1093/genetics/iyae051)
Supplement: iyae051_Supplementary_Data [file iyae051_supplementary_data.zip › Supplemental_Figure_7_GENETICS-2024-306814.pdf]

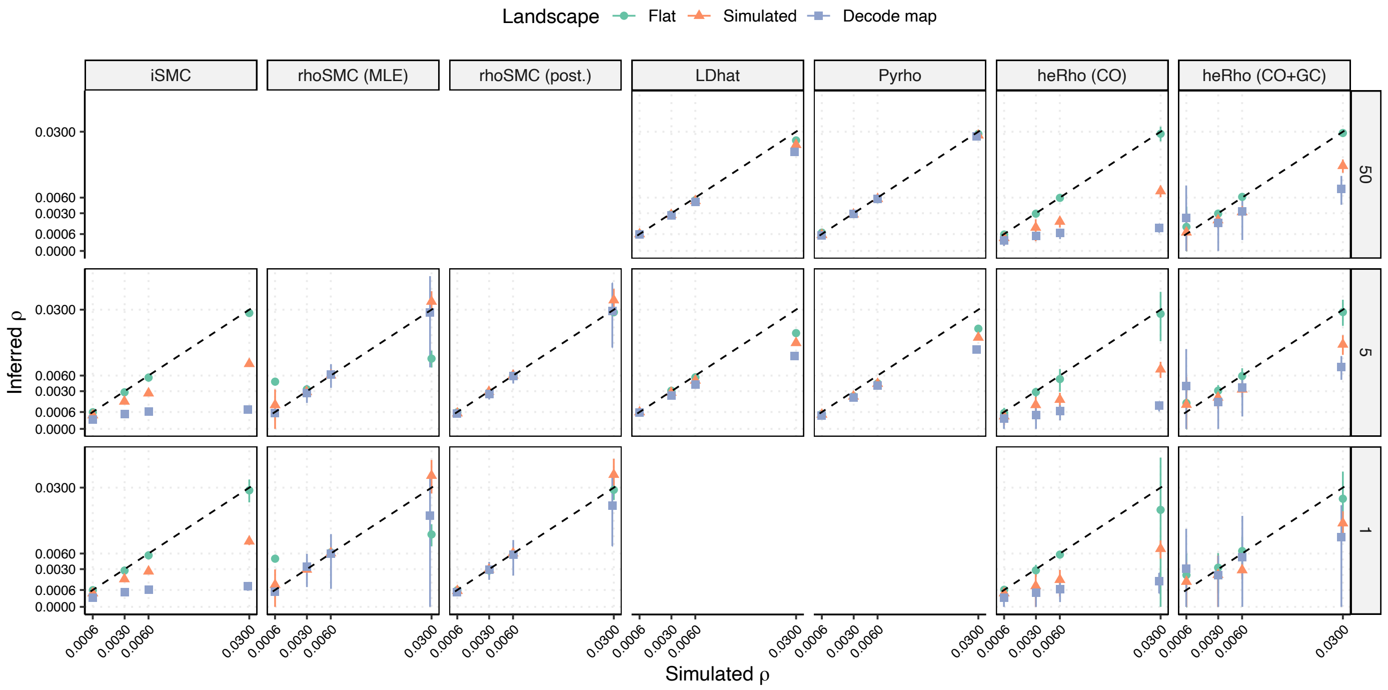

Supplementary Figure 7 Inference of the genome-wide population recombination rate under constant population size and various recombination landscapes, including a flat landscape, a simulated auto-correlated landscape, and a section of the DECODE recombination map. Legend as in Figure 2.
