# Supplementary material for: On the estimation of genome-average recombination rates
Source: Genetics. 2024 Apr 3;227(2):iyae051. doi: 10.1093/genetics/iyae051 (PMC11232287; doi:10.1093/genetics/iyae051)
Supplement: iyae051_Supplementary_Data [file iyae051_supplementary_data.zip › SupplementaryFigure5.pdf]

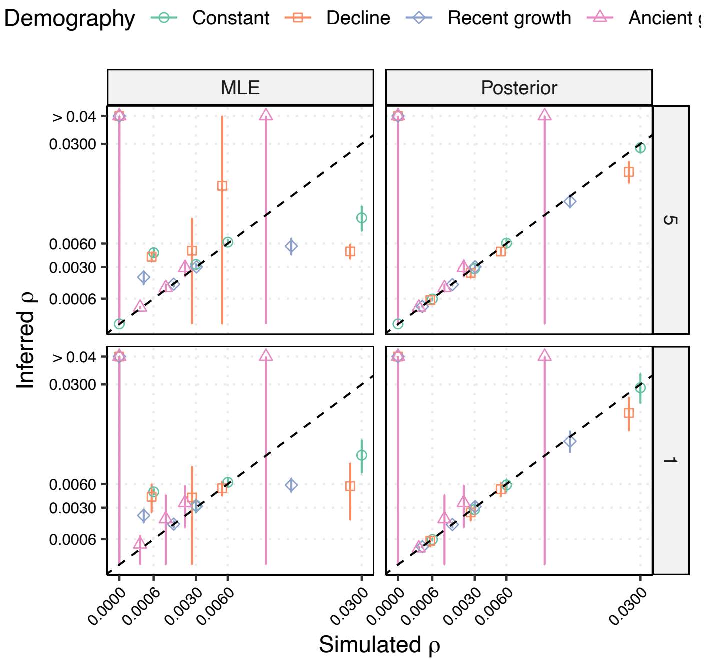

Supplementary Figure 5 Performance of the *rhoSMC* method when the simulated recombination landscape is homogeneous. MLE: maximum likelihood estimates. Posterior: posterior average estimates. Facet rows show the sample size (number of diploid individuals).
